# The Texas collaborative center for hepatocellular cancer: Reducing liver cancer mortality in Texas through coordination, collaboration and advocacy

**DOI:** 10.3389/fonc.2022.953933

**Published:** 2022-08-19

**Authors:** Ariel C. Harrison, Fasiha Kanwal, Sumeet K. Asrani, Aaron P. Thrift, Chris I. Amos, Maria L. Jibaja-Weiss, Jane R. Montealegre, Jessica P. Hwang, Amit G. Singal, Hashem B. El-Serag

**Affiliations:** ^1^ Department of Medicine, Baylor College of Medicine, Houston, TX, United States; ^2^ Department of Medicine, Baylor Scott and White, Dallas, TX, United States; ^3^ School of Health Professions, Baylor College of Medicine, Houston, TX, United States; ^4^ Department of Pediatrics, Baylor College of Medicine, Houston, TX, United States; ^5^ Department of General Internal Medicine, The University of Texas MD Anderson Cancer Center, Houston, TX, United States; ^6^ Department of Medicine, University of Texas Southwestern Medical Center, Dallas, TX, United States

**Keywords:** collaborative center, Texas, hepatocellular cancer (HCC), liver cancer, cancer prevention

## Abstract

Texas has the highest age-adjusted incidence rate of hepatocellular carcinoma (HCC) in the United States. To address cancer prevention and early detection through research, Cancer Prevention and Research Institute of Texas (CPRIT) has funded the Texas Collaborative Center for Hepatocellular Cancer (TeCH) to facilitate liver cancer research, education and advocacy activities. This paper describes the organizational structure, program measures, the actions completed and future plans of TeCH. This center is comprised of several cores and committees including the Administrative Core, Steering Committee, Data and Biospecimen Core, Scientific Committee, Clinical Network Committee, and the Community Outreach Committee. Each core and committee provide its own level of connectivity and necessary research support. We have developed and published a TeCH Framework, a conceptual model designed for improving primary and secondary prevention of HCC. TeCH and its committees facilitate connections and collaborations among HCC researchers and clinicians, healthcare leaders, biotechnology companies and the public to reduce liver cancer mortality in Texas by 2030.

## Introduction

Hepatocellular carcinoma (HCC), the most common cancer of the liver, is the fastest rising cause of cancer-related deaths in the United States (U.S.) (1). Texas has been reported to have the highest age-adjusted incidence rate of HCC within the nation (2). A combination of rising prevalence of metabolic (dysfunction) fatty liver disease (MAFLD) and high prevalence of hepatitis C infection and alcoholic liver disease is likely to explain these trends (3). The 5-year survival of patients with HCC remains dismal at less than 15%, with cure possibly in a small proportion of patients who are detected at an early stage and receive transplant or surgical resection. The expression of immune checkpoint molecules, such as programmed death-1 (PD-1), cytotoxic T-lymphocyte antigen 4 (CTLA-4), lymphocyte activating gene 3 protein (LAG-3), and mucin domain molecule 3 (TIM-3) on tumor and immune cells as well as the high levels of immunosuppressive cytokines induce T cell inhibition and represent a major mechanism of HCC immune escape. The recent use of immune checkpoint inhibitors, as single agents or in combination with kinase inhibitors, anti-angiogenic drugs, chemotherapeutic agents, and locoregional therapies, offers great promise in the treatment of HCC especially among patients with preserved liver function and small or non-metastatic tumors (4). Therefore, focus on prevention and early detection is key in reducing the burden of HCC.

To address overall cancer prevention and early detection through research, the state of Texas has established the Cancer Prevention and Research Institute of Texas (CPRIT). Within this state legislated organization, the Collaborative Action Program (CAP) has been established to be a leader in efforts to reduce liver cancer burden in Texas by reversing the trajectory of HCC incidence and mortality. To maximize the impact of CAP, the Texas Collaborative Center for Hepatocellular Cancer (TeCH) was created to facilitate CAP research activities, foster cross-project collaborations and disseminate emerging findings. The specific objectives of TeCH include (1) connect and facilitate collaboration among researchers and help them make discoveries, (2) recruit Texas healthcare providers and systems to turn discoveries into actions that providers can take to prevent HCC and implement into their practices, and (3) create a panel of Texas community leaders to swiftly administer usable information to the public. In this paper, we describe the structure, the functions, program measures and the future plans of TeCH, which can serve as a model for similar cancer prevention networks.

## Methods

Funded and started in August 2019 based within Baylor College of Medicine, TeCH supports and enhances research collaborations among CAP researchers through an organizational structure of several cores and committees. We have several process and outcome measures. The process measures are described with each of the committees and cores. Our overall lead outcome measures (i.e., intermediate outcomes) include the implementation of a state wide Texas Viral Hepatitis Elimination Plan, a NASH Awareness Campaign, implementation of EHR based screening and FIB-4 calculation to increase the detection of patients with advanced fibrosis and cirrhosis, and providing feedback to CPRIT about targeted research funding. Our ultimate outcome measure is decreasing HCC related incidence and mortality in Texas by 2030.

The Administrative Core houses infrastructure to facilitate and coordinate TeCH activities including monitoring CAP research, fostering cross-project collaboration, and disseminating emerging findings. This core also organizes the annual TeCH Symposium which targets researchers and healthcare providers. The Steering Committee convenes the membership of all CAP Principal Investigators (PI) to share their progress with leaders of TeCH Committee and Cores. The Scientific Committee selects and invites few prominent HCC researchers and thought leaders for a yearly meeting to identify and discuss with TeCH the potential connections among CAP projects, and provide general insights into science and practice of HCC prevention. To create an efficient environment that supports data sharing and collaboration, the Data and Biospecimen Core is comprised of experts in data harmonization and statistical analyses who support data storage and sharing for the CAP projects. This core also serves as a data repository for the CPRIT-funded Texas HCC Consortium (THCCC), the largest known prospective cohort of patients with cirrhosis (5). Recruitment of patients with cirrhosis began December 21, 2016 from five institutions in three cities (Houston Veterans Administration and Baylor Clinic in Houston; University of Texas Southwestern and Parkland Hospital in Dallas; and University of Texas San Antonio in San Antonio) and then expanded to McAllen, Texas in October 2019. Clinical history, risk factor questionnaires, liver imaging, laboratory data, and research blood samples were collected at enrollment and at each 6-month follow-up visits until HCC development or death. The Clinical Network Committee is comprised of clinician representatives from several large Texas healthcare systems and practices as well as payers and implementation specialists, and serves as a platform to disseminate HCC research advances along with disseminating educational material and clinically actionable recommendations to the front line clinicians and healthcare providers. This committee also facilitates the translation of innovations into clinical practice. Lastly, the Community Outreach Committee promotes and facilitates the engagement of community stakeholders in HCC research, supports community outreach and engagement initiatives, and facilitates the dissemination of research findings, and clinical and public health guidelines to the public. This committee also produces and collates culturally sensitive educational material on HCC risk factors, prevention, diagnosis and treatment targeted at providers, patients and at-risk communities for dissemination by community partners.

## Results

**Figure 1:**
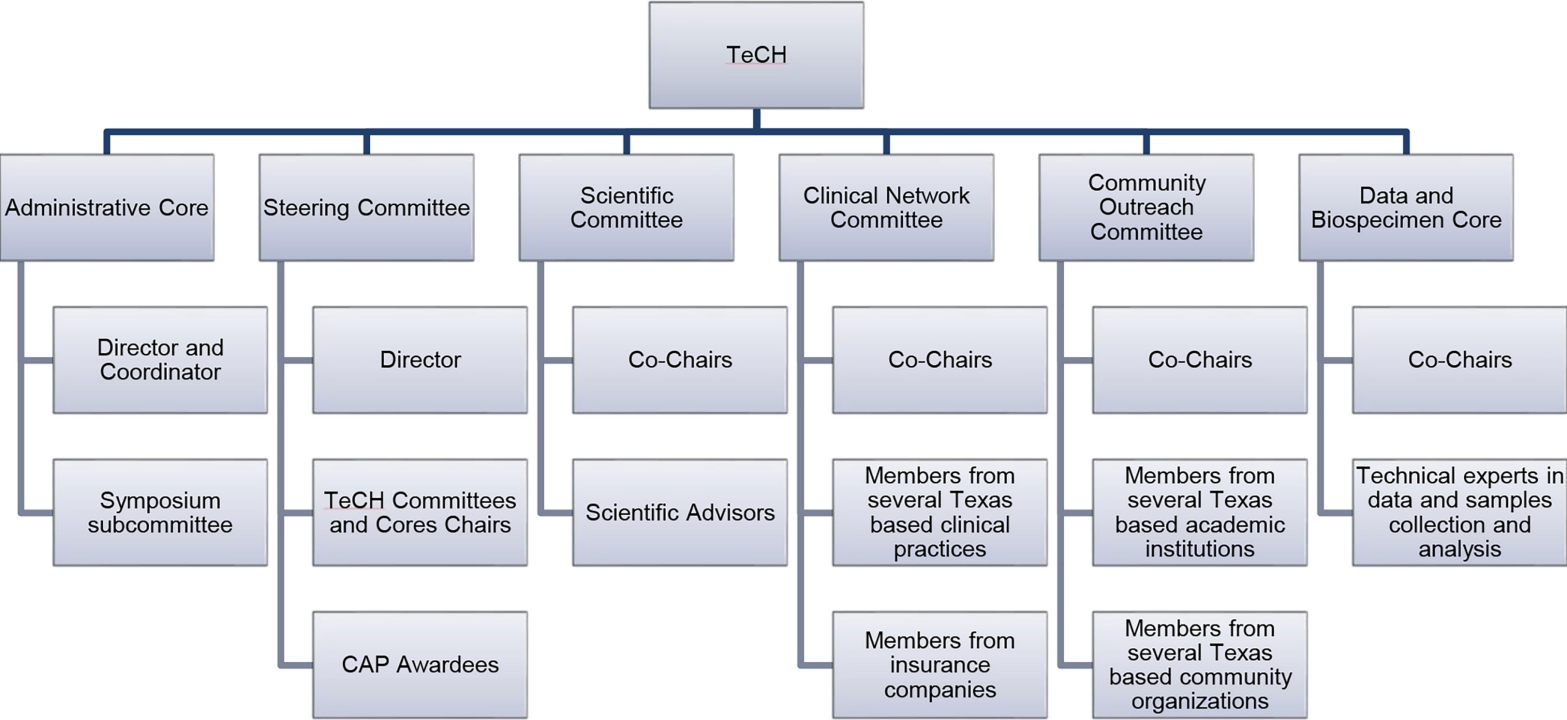
The Committees and Cores of the TeCH.

The different committees and cores of TeCH are shown in **Figure 1**.

### Administrative core

This Core has provided administrative support for the TeCH Committees and Cores, and Symposia including coordinating meetings, developing and maintaining the TeCH website (https://www.bcm.edu/research/research-centers/texas-collaborative-center-for-hepatocellular-cancer), and planning the Annual Symposium. To date, this core has successfully organized two TeCH Symposia, the first in October 2020 and the second in October 2021. The agenda for the first symposium included topics including HCC epidemiology, risk factors, and prevention including surveillance with Texas-based HCC researchers who presented current research. The second symposium included basic and translational science topics in HCC pathogenesis, prevention and treatment. CPRIT funded researchers as well as renowned HCC researchers from across the U.S. presented their current research at both symposia. The third annual symposium is being planned for September 2022, and it will be geared toward “From Research to Policy”. It will involve multi-stakeholders including policy makers, advocacy groups, payors, and healthcare organization leaders and administrators and will be organized in collaboration with the Rice University’s Baker Institute for Public Policy. We have learned in the process of planning the latter symposium that we would have benefited from convening an advocacy and policy committee since the inception of TeCH. The structure, focus and deliverable of the talks and meeting differed markedly from our traditional scientific meetings.

The Director of this core also fields questions and inquiries from prospective applicants to CPRIT HCC prevention grants. This service also included a liaison function by connecting to other investigators and/or repositories for relevant samples. This function, which was neither anticipated nor described in the application, has proven both popular and useful and in retrospect should have been offered, automated and marketed more.

### Steering committee

The Steering Committee has convened quarterly meetings since its inception in 2019. The Steering Committee is directed by the TeCH PI, consists of the PIs of the four CPRIT CAP Research Awards and the leaders of the other TeCH Committees. The quarterly meetings facilitate communication and interaction across the CAP research projects. This committee also reviews and provides guidance about the scientific progress of the CAP research projects through a rotation of presentations by the PIs, addresses administrative or scientific challenges, approves funding for pilot studies, and reviews and approves annual TeCH reports.

### Scientific committee

This committee has invited seven prominent experts in HCC research members from various institutions including Mayo Clinic, Mount Sinai, University of California Davis, and Harvard University. The members cover disciplines and areas of expertise of basic, translational, and clinical sciences associated with prevention, early detection, diagnosis, and treatment of HCC. This committee’s biannual meeting is structured for invited participants to prepare and deliver a 5-minute update on one breakthrough discovery or research direction, followed by a discussion of the scientific discoveries from CAP awards and the larger HCC field. The Scientific Committee also recommends collaborative studies to CAP PIs, topics and speakers for the Annual Symposium, and research ideas and directions to the Steering Committee. The committee also discusses and ranks the likely most influential research studies including clinical trials that are likely to facilitate the reduction of HCC mortality. These are then shared as recommendations for funding by CPRIT. We subsequently learned that this group was well suited to serve as the external advisory committee for TeCH, and implemented this change in Year 3.

### Data and biospecimen core

This Core serves as the centralized resource for the CAP projects because this core also functions as a data coordination center as well as data and biospecimen repository for the Texas HCC Consortium (THCCC) (5, 6). The THCCC cohort has been designed to facilitate the conduct of prospective-specimen-collection, retrospective-blinded-evaluation (PRoBE) design in which biologic specimens are collected prospectively from a cohort that represents the target population that is envisioned for clinical application of the biomarkers for the early detection of HCC (i.e., tertiary prevention) (7). The core assists in the design of studies and seeks to facilitate the development of research that impact risk for liver cancer or its treatment. We subsequently learned that unlike a traditional data coordinating center for a single, this core could not serve this function for multiple studies that were not designed with this core in mind and each brought its own logistical and regulatory complexities. Therefore, this core was limited to a consultative role for data sharing agreements, data linkages, and facilitating collaborations.

### Clinical network committee

**Figure 2:**
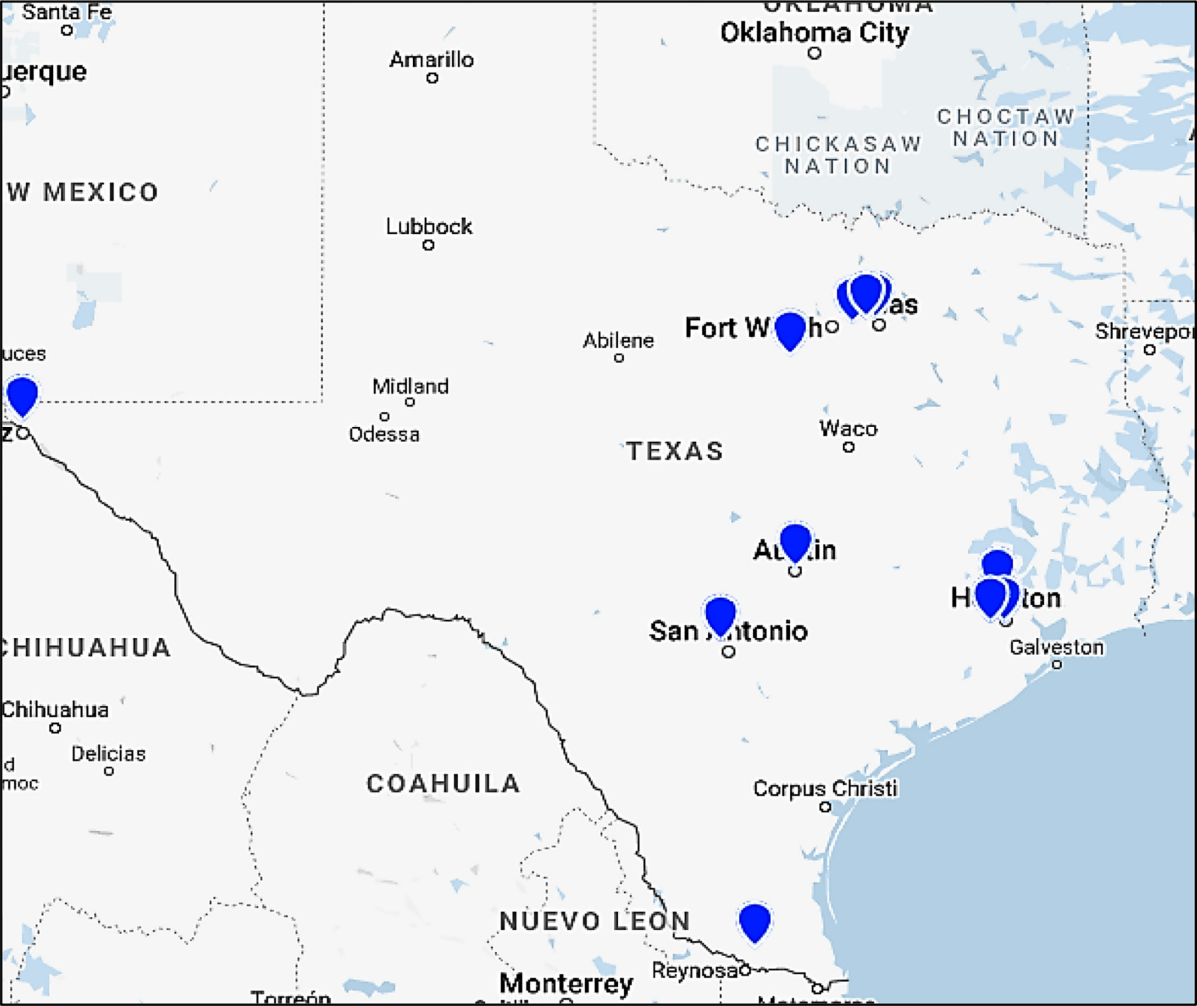
Map of the TeCH Clinical Network Committee Texas institutions.

The Clinical Network Committee has assembled an extensive state-wide clinical network with broad representation including primary and specialty care providers, healthcare organizations, and large insurance carriers. Members of this committee represent organizations such as United Healthcare (UHC), Kelsey-Seybold, Optum, Athena, Centene, Baylor Scott and White Health Dallas and Temple, The University of Texas MD Anderson Cancer Center, UT Southwestern Medical Center, BCM, and others (**Figure 2**). The subcommittees and the leaders of this committee have met nine times throughout 2021. This committee was initially too large to be nimble. The leader of this committee has divided this committee in Year 2 into three subgroups: (1) Insurance, (2) IT professionals, clinical informatics and physicians, and (3) Patient and Provider Material Development.

**Figure 3:**
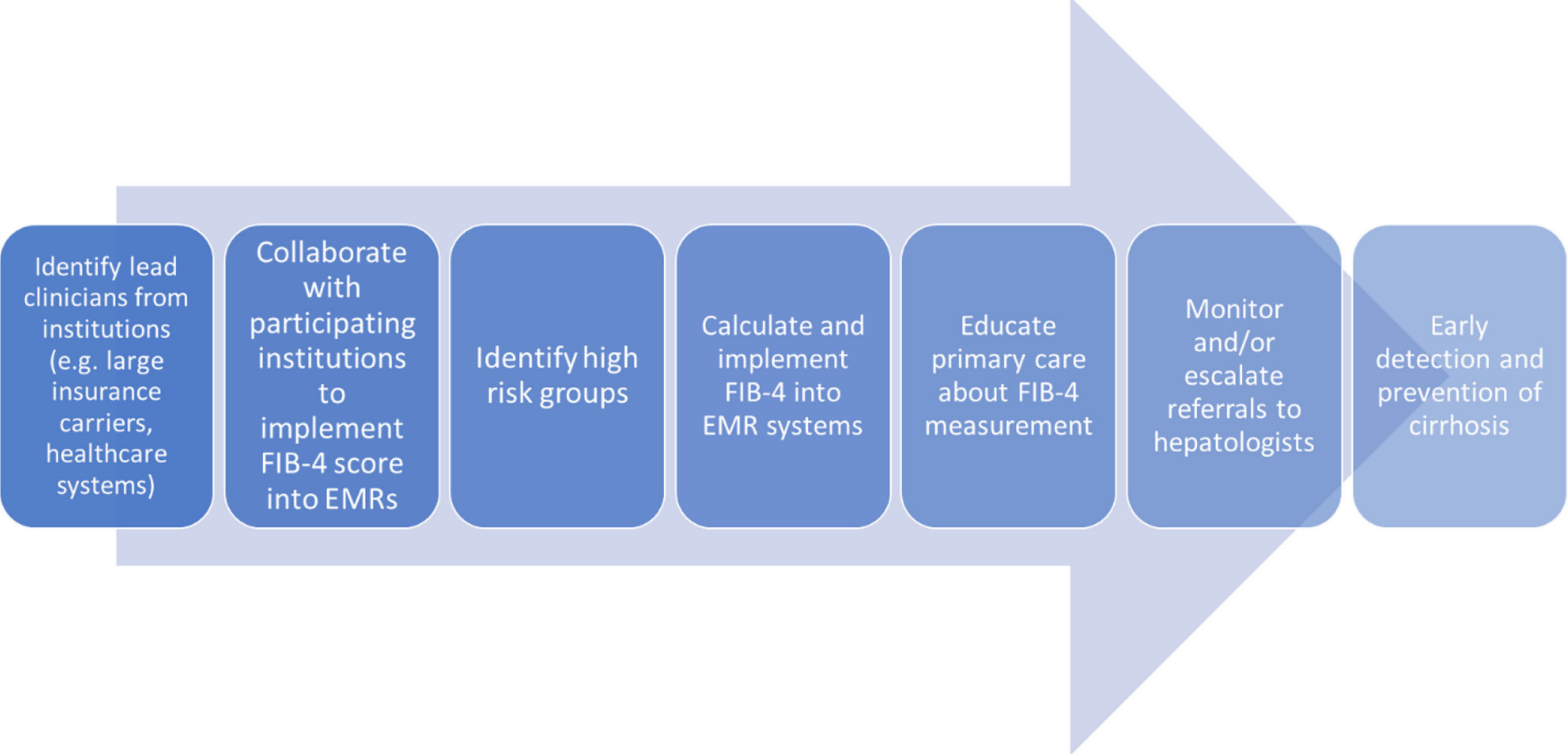
Process of FIB-4 Implementation at various healthcare systems.

Another major learned lesson has been in the way this committee has identified and ranked based on effectiveness, impact and feasibility several possible clinical interventions targeting HCC prevention. Based on this ranking system, the first priority was determined to be implementing a scoring system known as the Fibrosis-4 (FIB-4) Calculator, designed to estimate the amount of liver fibrosis, into the electronic medical records of several hospitals in the network. The committee have identified healthcare partners who have either implemented automated calculation of FIB-4 with or without clinical care pathways into their EMRs or are in the process of doing so (**Figure 3**). FIB-4 implementation would facilitate, by identifying high risk groups with advanced hepatic fibrosis, both secondary HCC prevention (chemoprevention, weight loss), and tertiary prevention (screening and surveillance). In the development phase, a variety of practices were examined showing that approximately 50% of patients at high risk of advanced fibrosis were not referred for specialty care. Currently, the committee is working on implementation of automated FIB-4 calculation in the electronic health record systems of three diverse settings within a large healthcare system (urban, rural and charity primary care practice) and surveying providers in the different settings of optimal way of implementation relevant to their practice.

### Community outreach committee

**Figure 4:**
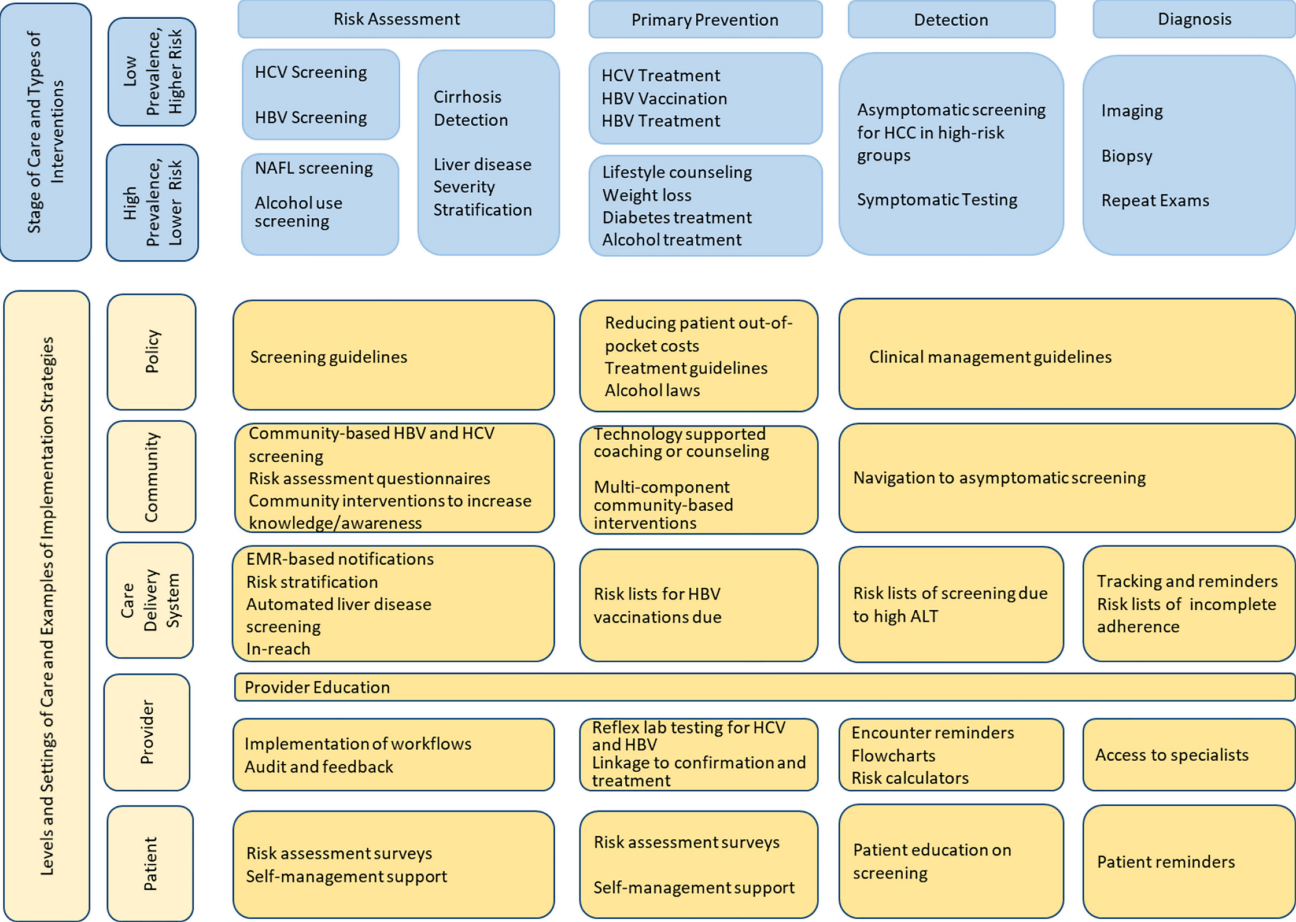
TeCH Framework: A conceptual model for improving primary and secondary prevention of HCC by focusing on implementation and evaluation of intervention strategies across the HCC care continuum.

The Community Outreach Committee is comprised of experts in prevention from across various Texas institutions including Baylor College of Medicine, University of Texas Health Science Center School of Public Health in Houston and in San Antonio, the University of Texas Southwestern Medical Center, as well as community representatives and leaders such as the Hope Clinic. This committee has also enlisted a patient advocate who will act as a representative of patients and families suffering from liver cancer, and ensure that the communities affected by liver cancer are heard by the committee and the leaders of TeCH. The co-leader of this Community Outreach Committee in collaboration with TeCH leadership has developed and published a TeCH framework (**Figure 4**), a conceptual model designed for improving primary, secondary and tertiary prevention of HCC by focusing on implementation and evaluation of intervention strategies across the HCC care continuum (8). Primary prevention focuses on detection and treatment of main HCC risk factors (viral hepatitis, unhealthy alcohol drinking, metabolic dysfunction). Secondary prevention focuses on detection and treatment of risk factors among patients with liver disease. Tertiary prevention involves screening and surveillance for HCC in high-risk groups (e.g., cirrhosis). This framework would serve as a blueprint for the different TeCH initiatives and a platform for subsequent measurement and research. We anticipate that as primary prevention of HBV and HCV related HCC continue to succeed through HBV vaccination, screening and HBV/HCV antiviral treatment, the HCC burden shift toward metabolic dysfunction fatty liver disease is likely to increase emphasis on secondary and tertiary prevention (i.e., increasing early detection to be treated by highly effective new treatments).

This committee has created a repository of educational materials about HCC risk factors (hepatitis B, hepatitis C, alcohol liver disease, cirrhosis and non-alcoholic fatty liver disease) from multiple sources and in various languages (Spanish, Vietnamese, among others), making the materials freely available on the TeCH website (https://www.bcm.edu/research/research-centers/texas-collaborative-center-for-hepatocellular-cancer/educational-resources). This material has been vetted for accuracy and contemporary relevance. In addition, the leaders of this committee are collaborating with biotechology companies to help create and disseminate liver cancer prevention resources to at-risk communities. There were 2 major learning points from this committee. We should have aligned and leveraged earlier than we did the efforts of several other established organizations and foundations that focus on community outreach for hepatitis B and C. We have started these efforts with mixed success in Year 3. The second leaning point is that while several members and actions of this committee focused on advocacy, we should have created a committee that is focused exclusively on advocacy and policy.

## Discussion

The objectives of TeCH are to connect and facilitate collaboration among researchers and assist them in conducting studies and making discoveries, recruit Texas healthcare providers and systems to turn discoveries into actions to prevent HCC and implement into their practices, and create a panel of Texas community leaders to swiftly administer usable information to the public. TeCH and the CPRIT CAP program that funds it represent a novel promising collaborative model for responding to the liver cancer burden for the state of Texas. The long-term goal of reducing HCC mortality by 2030 will be achieved through accomplishing several process and intermediate outcome measures. Unique features include the state (Texas) focus, the broad coordinating features (distinct from data coordinating centers), the dissociation from initial review and funding decisions, and translational focus of its mission (translation to practice and policy). Given the CPRIT funding, only Texas based institutions can join, however individual investigators are welcome to join from anywhere.

In addition to THCCC, TeCH currently supports four active CPRIT CAP funded studies (5, 6). Dr. Jessica Hwang at The University of Texas MD Anderson Cancer Center leads one of the CAP research projects, designed to identify the best way to screen patients at risk for fibrosis and cirrhosis, and to help these patients manage risk factors. This study will identify feasible, efficient, and cost-effective ways to assess patients in a primary care setting for the major risk conditions that can lead to fibrosis or cirrhosis. This study will also provide evidence to support a screening strategy for NAFLD which currently has no screening strategy. Further, this project has the potential to change the field of liver cancer research by providing primary care providers evidence-based screening and management strategies to detect and treat medical conditions that are risk factors for fibrosis and/or cirrhosis.

Dr. Aaron Thrift at Baylor College of Medicine leads another CAP research project that is designed to study genetic risk factors for HCC among Hispanics in Texas. This research project was identified as high priority as Hispanics in Texas, especially Hispanics living in South Texas and U.S./Mexico border regions, who now have the highest incidence rates for HCC in the nation. However, the reasons for this high burden of HCC are not known. This study will help identify factors that predispose Hispanics to high HCC risk and help with efforts to prevent HCC in this high-risk and underserved population. TeCH and the THCCC were involved in the development of this study and have helped the investigators with connection to study sites necessary for patient recruitment and data and biospecimen collection and storage.

Dr. Amit Singal at UT Southwestern leads the third CAP research project focused on evaluating a blood-based biomarker for risk stratification and early detection of HCC. This study is also conducting phase III validation of two biomarker panels for early HCC detection – GALAD and Glycotest – to see if these offer high sensitivity for early-stage HCC. The hypothesis for this study is that androgens increase risk of HCC and estrogens decrease risk of HCC. Further, this proposal leverages data and samples from THCCC to evaluate the extent to which circulating sex hormones are associated with HCC risk in men and women in a nested case-control study.

Lastly, Dr. Fasiha Kanwal at Baylor College of Medicine leads a CAP research project focused on understanding the mechanisms underlying the racial/ethnic and socioeconomic disparities in progression to HCC. In a large, multicenter, racially and socioeconomically diverse cohort of patients with cirrhosis treated in four healthcare systems, this project will use causal mediation analyses to determine the contribution of factors at the individual, interpersonal and community levels to disparities in HCC. We will leverage stored genomic data for a subset of patients enrolled in THCCC to examine the contribution of liver disease genetic variants (*e.g*., *PNLPA3*) to disparities in HCC and related outcomes. If successful, this study will produce valid, generalizable results that offer insights into *why* disparities exist. It will also identify actionable targets for interventions aimed at improving HCC outcomes and reducing disparities.

During two CPRIT research grants submission cycles in 2020 and 2021, TeCH PI and administrators have addressed multiple queries, wrote support letters, and in some instances connected investigators to resources or other investigators. TeCH investigators also had successful submission for program project support from CPRIT and from the National Cancer Institute; the proposed studies leverage THCCC, TeCH and address methods of risk stratification and chemoprevention of HCC among patients with MAFLD.

This center aims to impact surrounding communities through disseminating patient and provider educational material to local clinics, collaboration with local organizations, implementation of liver fibrosis scoring systems into EMRs, and influencing health and public health policy. TeCH also aspires to collaborate with researchers and other professional societies outside of Texas to improve on the ultimate goal of reducing liver cancer mortality. TeCH and its committees are also facilitating connections with primary care and frontline providers, HCC researchers, healthcare leaders, biotechnology companies and the public to reduce liver cancer mortality in Texas.

## Data availability statement

The original contributions presented in the study are included in the article/supplementary material. Further inquiries can be directed to the corresponding author.

## Author contributions

HE-S contributed to the conception of the paper funding, editing and writing. AH wrote the first draft of the manuscript. FK, AT, JH, and AS provided descriptions of their studies. FK, SA, AT, CA, MJ-W, JM, JH, and AS contributed to manuscript revision. All authors contributed to reading, revisions, and approval of the submitted version.

## Funding

The work is funded by Cancer Prevention Research institute of Texas (CPRIT) award and in part by NIH P30DK56338 to Dr. El Serag.

## Conflict of interest

The authors declare that the research was conducted in the absence of any commercial or financial relationships that could be construed as a potential conflict of interest.

## Publisher’s note

All claims expressed in this article are solely those of the authors and do not necessarily represent those of their affiliated organizations, or those of the publisher, the editors and the reviewers. Any product that may be evaluated in this article, or claim that may be made by its manufacturer, is not guaranteed or endorsed by the publisher.
